# Qualitative assessment of the suitability of the Dysphagia Symptom Questionnaire to monitor dysphagia in children aged 7–10 years with eosinophilic esophagitis

**DOI:** 10.1186/s41687-023-00646-z

**Published:** 2023-10-31

**Authors:** Robin M. Pokrzywinski, Bridgett Goodwin, Evan S. Dellon, Ellyn Kodroff, Anne Brooks, Adam Bailey, James Williams, Nirav K. Desai

**Affiliations:** 1 Evidera, 7101 Wisconsin Ave #1400, Bethesda, MD 20814 USA; 2 Takeda Development Center Americas, Inc., Cambridge, MA USA; 3 Center for Esophageal Diseases and Swallowing, Division of Gastroenterology and Hepatology, Department of Medicine, University of North Carolina at Chapel Hill, Chapel Hill, NC USA; 4 Campaign Urging Research for Eosinophilic Disease, Lincolnshire, IL USA

**Keywords:** Dysphagia, Qualitative assessment, Patient-reported outcomes, Esophageal eosinophilia, Pediatric, Electronic device

## Abstract

**Background:**

The Dysphagia Symptom Questionnaire (DSQ) is a patient-reported outcome measure that assesses the frequency and severity of dysphagia in patients with eosinophilic esophagitis (EoE); however, it has only been validated for use in patients with EoE aged 11–40 years. This study examined the content validity of the DSQ and its usability on an electronic handheld device in children aged 7–10 years with EoE.

**Methods:**

In this qualitative, observational cohort study, participants were recruited to partake in two rounds of interviews. During visit 1, a cognitive interview examined EoE-associated concepts and the appropriateness of the DSQ for assessing dysphagia. Participants completed the DSQ daily for 2 weeks, and DSQ scores were calculated. After 2 weeks, a second interview assessed the usability of the DSQ on the electronic device and the burden associated with completing it daily.

**Results:**

Overall, 16 participants were included (aged 7–8 years: n = 8; aged 9–10 years: n = 8); most were male (75%) and white (81%), and the mean (standard deviation [SD]) age was 8.4 (1.3) years. The most commonly reported EoE-associated concept was ‘trouble with swallowing’ (63% [10/16]). Most participants reported that the questions were ‘easy to complete’ and ‘relevant to someone with EoE and dysphagia’. Overall, participants reported understanding the questions and associated responses; however, further probing demonstrated inconsistent comprehension. Key challenging concepts included ‘solid food’, ‘trouble swallowing’, ‘vomit’, and ‘relief’; some participants also reported difficulty differentiating between pain levels (31% [4/13]). Most caregivers reported that their child had experienced dysphagia (94% [15/16]); however, mean (SD) DSQ scores over the study period were low (7.3 [7.4]), suggesting infrequent and mild dysphagia, or a lack of comprehension of the questions. Most participants reported that completing the DSQ on the electronic device was easy (93% [14/15]) and they would be willing to complete it for longer than 2 weeks (73% [11/15]).

**Conclusions:**

Difficulties with comprehension and comprehensiveness suggest that the DSQ may not be sufficiently comprehensive for use in all patients in this population, and wording/phrasing changes are required before use in a clinical trial setting.

**Supplementary Information:**

The online version contains supplementary material available at 10.1186/s41687-023-00646-z.

## Background

Eosinophilic esophagitis (EoE) is a chronic, localized, immune-mediated esophageal disease, characterized clinically by symptoms of esophageal dysfunction and histologically by eosinophilic inflammation (≥ 15 eosinophils per high-power field) [1, 2]. Difficulty swallowing, or dysphagia, is one of the most commonly reported symptoms of EoE [2, 3]; however, signs and symptoms of EoE can vary by age [4]. In children, symptoms may be wide-ranging and include feeding problems, nausea and vomiting, abdominal pain, and heartburn, whereas adolescents and adults typically present most frequently with dysphagia and food impaction [2, 4, 5].

Given that dysphagia is considered a key feature of EoE, it is often used as a symptom outcome in clinical trials assessing therapeutic agents for this disease [5, 6]; therefore, owing to discordance between histologic and symptom outcomes, reliable, validated symptom measures are required [7]. As a result, the Dysphagia Symptom Questionnaire (DSQ), a patient-reported outcome (PRO) measure, was designed specifically to assess the frequency and severity of dysphagia in patients with EoE [6, 7]. The development of the DSQ was based on interviews with adolescents and adults with EoE to elicit symptoms and concepts associated with EoE [7]. The DSQ has been assessed for content validity and was psychometrically validated for use in adolescents and adults aged 11–40 years [6, 7]. It uses a 24-h recall period and comprises four questions assessing: solid food consumption (question 1 [Q1]); dysphagia frequency and severity (questions 2 and 3 [Q2 and Q3], respectively); and odynophagia (pain with swallowing) (question 4 [Q4]) [6, 7]. Q4 of the DSQ is considered an exploratory stand-alone item that can be scored as a separate concept. This is because not all patients with EoE reporting experiencing pain with swallowing [8], and in light of the results of interviews with adolescents and adults, who did not emphasize pain as an important symptomatic factor in EoE [6].

Clinical studies have used the DSQ to assess the impact of treatment with pharmacologic therapies on symptoms of dysphagia in adolescents and adults with EoE [9–13]. The psychometric properties of the DSQ have also been evaluated in patients aged 11–40 years with EoE using data from a 12-week, phase 2, randomized, placebo-controlled trial of budesonide oral suspension [6]. This psychometric analysis demonstrated that the DSQ is a valid and reliable measure of dysphagia in patients with EoE, thus supporting its use in clinical trials [6]. Given that the DSQ has not been evaluated in children with EoE aged younger than 11 years, and because there are limited symptom metrics for pediatric patients with EoE, it is important to determine whether it is an appropriate measure of dysphagia in this population for potential future studies and clinical trials.

We investigated the content validity and comprehensibility of the DSQ in children aged 7–10 years with EoE, and the usability of the DSQ on an electronic handheld device in this patient population. Content validity in this study was defined as the evidence demonstrating that the items of a PRO instrument are appropriate and comprehensive for its intended concept, population and use [14]. Comprehensibility was defined as the participant understanding the phrasing used in the DSQ as intended [15].

## Methods

The manuscript has been written in accordance with the Strengthening the Reporting of Observational studies in Epidemiology (STROBE) guidelines for reporting observational studies [16].

### Study design and recruitment process

**Fig. 1:**
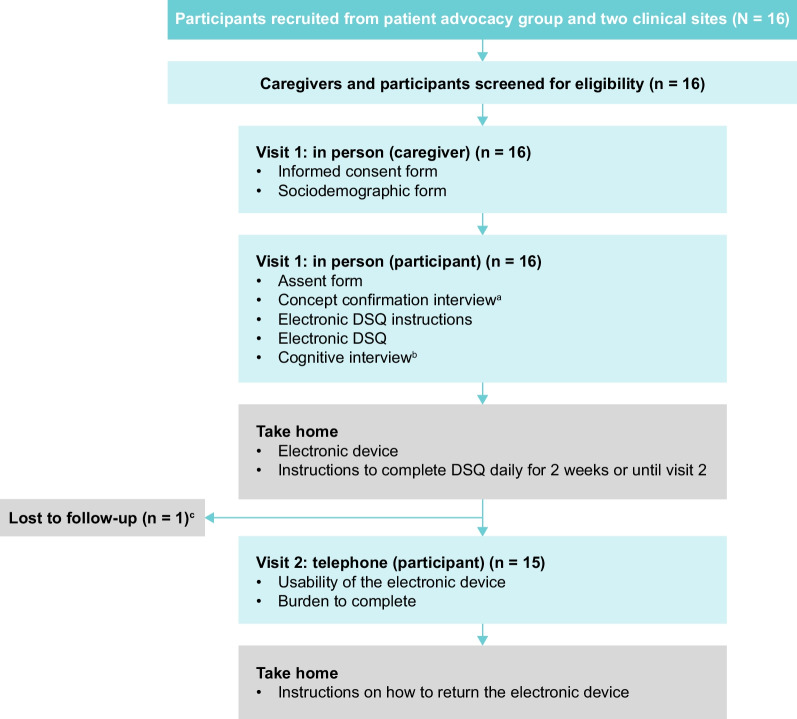
Study design and participant selection for caregivers and participants aged 7–10 years with EoE. ^a^To elicit concepts related to signs and symptoms of EoE. ^b^To assess DSQ instructions, completeness, relevance, comprehensibility of the content, the 24-h recall period, and response options. ^c^Contact was attempted three times before the participant was considered lost to follow-up. *DSQ* Dysphagia Symptom Questionnaire, *EoE* Eosinophilic esophagitis

This was a 2-week, cross-sectional, qualitative, observational, phenomenological study that used one-to-one cognitive interviews with children aged 7–10 years with EoE (herein referred to as ‘participants’) and their caregivers. The study design is shown in Fig. 1. Participants and their caregivers were recruited between March 5, 2019 and May 31, 2019, from the patient advocacy group Campaign Urging Research for Eosinophilic Disease (CURED) and two clinical sites in the USA (Rocky Mountain Pediatric Gastroenterology [Denver, CO] and Clinical Research of Charlotte [Charlotte, NC]). Participants and their caregivers who were recruited from CURED were part of their patient database. Recruitment was carried out via email or social media using institutional review board-approved materials; caregivers who responded were subsequently directed to Evidera, the research organization who conducted the qualitative research and subsequent data analyses, for screening and eligibility assessments (Fig. 1). All recruitment procedures complied with current Health Insurance Portability and Accountability Act regulations. Institutional review board approval of the study protocol (EVA-21524-01; February 2019; version 8.0) was obtained from the Ethical and Independent Review Services before participant recruitment, and informed consent and assent were obtained from each caregiver and participant, respectively, before conducting the first interview. This study adhered to guidelines from the International Society for Pharmacoeconomics and Outcomes Research on pediatric PRO instruments for research [17]. Before recruitment, all participants and caregivers were screened via telephone or in-person (at the US clinical study sites) using a standardized script to ensure the study was presented in a consistent manner. The US Food and Drug Administration (FDA) draft of the patient-focused drug development guidelines (guidance 3) were followed during the development of the protocol for this study [18].

### Study participants

To be eligible, patients (male or female) had to be aged 7–10 years at the time of screening with a clinician-confirmed diagnosis of EoE based on a caregiver report; be able to read, speak and understand English; agree to participate in telephone and in-person interviews (audio recorded) and complete study questionnaires in English; and provide assent and have a caregiver provide consent to participate in the study. Key exclusion criteria were: children with EoE requiring a feeding tube; and children with EoE exhibiting any clinically relevant and/or serious chronic medical conditions that may confound reports of signs and symptoms of EoE (e.g. malignancy, history of organ transplantation, congenital abnormalities, or other gastrointestinal disorders, such as Crohn’s disease), or interfere with their ability to participate in an interview and/or complete the study procedure (e.g. visual problems, severe mental illness, or cognitive impairment). The sample size in this study was based on the number of participants required to reach concept saturation; therefore, no sample size calculations have been provided.

### Interview process and data collection

The interview process is detailed below and shown in Fig. 1. As per standard practice for interviews with participants aged younger than 18 years, the caregiver was allowed to be present if desired, but was asked not to make any comments [17]. Interviews were conducted by scientific personnel trained in qualitative interview techniques and, specifically, in the cognitive interview guide, the DSQ and interviewing children [7]. For both participants and their caregivers, a sociodemographic form was completed. Each participant was paid US$100 via a prepaid card for their involvement in the full study; participants received US$75 for completing visit 1 and an additional US$25 for completing visit 2.

#### Visit 1

During visit 1, a cognitive interview was conducted by one of two interviewers to elicit concepts regarding signs and symptoms associated with EoE, determine whether the DSQ (version 4.0) was an appropriate measure of dysphagia and assess whether the electronic handheld device was suitable for self-administration of the DSQ in this patient population (Fig. 1). The complete cognitive interview guide and examples of questions on concept elicitation as well as probing questions are provided in the Additional file 1. The duration of the interviews was between 30 and 60 min, and they were completed in person either at the clinical study site (for participants recruited from the clinical study sites) or at the participant’s home, where two interviewers attended (for participants recruited via the patient advocacy group). The interview began with an overall explanation of the process, including instructions on how to use the electronic handheld device to complete the DSQ. The interview included a brief concept elicitation discussion with the participant about their signs and symptoms related to EoE. Participants (children aged 7–10 years) then completed the DSQ. After the interview, participants were provided with the electronic handheld device and instructed to complete the DSQ daily over 2 weeks.

#### Visit 2

A 30 min telephone interview was conducted at visit 2, which was scheduled to take place 2 weeks after visit 1 (Fig. 1). Its purpose was to ask participants about the usability of the electronic handheld device (e.g. difficulties in completing the diary) and their perceptions of the burden associated with diary completion. Procedures to return the device were also explained at this visit.

### Interview guide and questions

A semi-structured interview guide, designed to maintain consistency across different interviewers, formed the basis of the cognitive interviews. The cognitive interview and usability component included questions about the relevance and importance of the individual DSQ items and the associated response options for this population (children with EoE aged 7–10 years). The cognitive interview also addressed any lack of clarity around items, terminology, instructions and the daily recall period for this population, considering that the DSQ was originally validated in patients with EoE aged 11–40 years [6]. Following participants’ responses to the items, qualitative interviewing techniques were employed to assess the readability, understandability, ease of completion, relevance, and comprehensiveness of the item, and the clarity of the wording and instructions for completion. These were particularly important to establish, given the young age of participants in this study. Participants were asked what they thought each item meant, how they interpreted the instructions and how they arrived at their responses. The interviewer also asked about the appropriateness of the response options for each question, the daily recall period, and the ability of participants to recall the information requested accurately. This aimed to further elicit comprehensibility of the DSQ in children aged 7–10 years with EoE. The interviewers were debriefed after their first few interviews to assess the performance of the interview guide and maintain consistency across interviews.

### Qualitative and quantitative assessments

The DSQ was specifically developed to enable quantitative measurement of dysphagia associated with EoE [7]; however, the focus of our study was qualitative assessment of participants’ understanding of the content of the DSQ and the usability of the electronic handheld device. The DSQ uses a daily recall period and comprises four questions on whether an individual has consumed solid food (Q1), the frequency (Q2) and severity (Q3) of solid-food dysphagia and the pain associated with swallowing (Q4) (Additional file 1: Table S1) [6, 7]. DSQ scores were calculated from the combined responses to Q2 and Q3, and could range between 0 and 84 for the 2-week study period, with lower scores indicating less frequent and/or less severe dysphagia [6]. A sociodemographic form was used to characterize the sample of enrolled children with EoE and their caregivers during visit 1. Responses were analyzed qualitatively and quantitatively. A content analysis approach was used to analyze the data (based on field notes, audio recordings, and transcripts) from the interview sessions. All qualitative analyses were performed using ATLAS.ti. Each transcript was independently coded by one scientific staff member and subsequently reviewed by a second scientific staff member for accuracy and completeness (analysis triangulation). Any coding discrepancies were resolved through consultation with Evidera, providing multiple perspectives on the outputs. Analyses were stratified by age group (aged 7–8 and aged 9–10 years). Qualitative analysis involved transcription and review of each interview audio recording followed by development of a coding dictionary based on the cognitive interview guide to organize and catalog participant themes from responses to questions, including concept elicitation, cognitive and usability portions of the interview. Codes from this dictionary were applied to participant text in each transcript. The coders communicated throughout the process regarding correct coding practices and provided input about adding further codes, based on the actual transcripts and their experience assigning the codes. Upon completion of the coding process and review, a narrative summary was developed that focused on comprehension of the DSQ. Sociodemographic data were entered in a DataFax system which generated automated queries for incomplete or conflicting data. Queries were resolved internally by Evidera. Sociodemographic data and responses to the DSQ were then exported into Statistical Analysis System software, version 9.4 [19], to perform quantitative analysis. Descriptive analyses (mean [standard deviation], or counts and frequencies) were used to summarize demographic and clinical characteristics.

## Results

### Demographics and baseline characteristics

#### Participants (children aged 7–10 years with EoE)

Participants were recruited between March 5, 2019 and May 31, 2019. In total, 16 participants (CURED, n = 5; two US clinical sites, n = 11) were considered eligible for inclusion; interviews were conducted between April 25, 2019 and July 2, 2019 (Table 1). Of the 16 participants, 15 attended visit 2 and one was lost to follow-up. The mean (standard deviation [SD]) age of participants was 8.4 (1.3) years. Most participants were male (75% [12/16]) and white (81% [13/16]). When asked if their child had ever experienced difficulty swallowing (i.e. ‘food getting stuck or going down slowly’), most caregivers (94% [15/16]) reported that their child had this symptom.

**Table 1:** Baseline demographics and clinical characteristics of children with EoE based on the caregiver report

Demographic/clinical characteristic	Children with EoE (caregiver report)		
	7–8 years (n = 8)	9–10 years (n = 8)	Total (N = 16)
Age, years			
Mean (SD)	7.3 (0.5)	9.6 (0.5)	8.4 (1.3)
Median (range)	7.0 (7.0–8.0)	10.0 (9.0–10.0)	8.5 (7.0–10.0)
Sex			
Male	5 (63)	7 (88)	12 (75)
Female	3 (38)	1 (13)	4 (25)
Ethnicity			
Hispanic or Latino	1 (13)	1 (13)	2 (13)
Not Hispanic or Latino	7 (88)	7 (88)	14 (88)
Race			
White	7 (88)	6 (75)	13 (81)
Other^a^	1 (13)	2 (25)	3 (19)
Education level			
Primary/elementary school^b^	7 (88)	7 (88)	14 (88)
Other^c^	1 (13)	1 (13)	2 (13)
Age at diagnosis of EoE, years, mean (SD)	4.9 (1.1)	4.9 (2.1)	4.9 (1.6)
Has experienced difficulty swallowing food (ever)	7 (88)	8 (100)	15 (94)
Currently receiving dietary therapy^d^	7 (88)	6 (75)	13 (81)
Received prior dietary therapy^e^	6 (75)	6 (75)	12 (75)
Frequency of EoE signs and symptoms exhibited by child			
Never	1 (13)	0 (0)	1 (6)
1–2 days per week	4 (50)	5 (63)	9 (56)
3–4 days per week	1 (13)	2 (25)	3 (19)
5–6 days per week	1 (13)	0 (0)	1 (6)
Every day	1 (13)	1 (13)	2 (13)

All data are reported as n (%), unless otherwise stated

^a^Other race includes: 'white and black/African American' (n = 2) and 'Latino' (n = 1)

^b^Primary/elementary school grades include: ‘1st’ (n = 1); ‘First Wesleyan’ (n = 1); ‘2nd’ (n = 3); ‘3rd’ (n = 1); ‘4th’ (n = 4); and ‘5th’ (n = 1). Three participants did not specify grade

^c^'Homeschool' (n = 2)

^d^Current dietary therapy reported by caregivers: 'avoiding dairy, chicken, turkey', 'no wheat and no dairy', 'no dairy, some nuts', 'soy, milk, peanuts, some tree nuts, gluten, eggs', 'gluten free', 'limitation', 'we avoid dairy (but not always) – reduced', 'strict avoidance (IgE): dairy, peanuts, tree nuts, eggs; twice daily swallowed budesonide slurry; once daily 30 mg PPI and not in remission', 'no dairy or wheat', 'elimination of egg, dairy, soy, almond, string beans', 'food elimination modified elemental', 'dairy, gluten, peanuts, tree nuts, bananas', and 'gluten, egg, dairy, soy, nuts'

^e^Prior dietary therapy reported by caregivers: 'avoiding chicken, dairy, turkey, wheat, corn, eggs', '1st dairy elimination for 3 months (2017, wheat elimination for 3 months and dairy 2018)', '2 years', 'ages 2–3, allergies at all, milk, wheat, etc.', 'last 5 years', 'same, avoid dairy (but not always) – reduced', 'since birth/infancy for IgE since age 4 for EoE’, ‘always’, ‘eliminating: dairy, wheat, egg, soy, peanut, fish/shellfish, all meat, potatoes, rice, corn’, ‘top 8, beef and chicken ‘2017–2018', and '01/2017'

*EoE* Eosinophilic esophagitis, *IgE* Immunoglobulin E, *PPI* Proton-pump inhibitor, *SD* Standard deviation

#### Caregivers

The mean (SD) age of caregivers was 37.1 (6.9) years (Additional file 1: Table S2). Most caregivers were female (94% [15/16]), white (69% [11/16]), and identified as the participant’s mother (94% [15/16]). In addition, most caregivers reported being married (81% [13/16]) and half (50% [8/16]) reported being employed full or part time.

### Concept elicitation – signs and symptoms of EoE

**Fig. 2:**
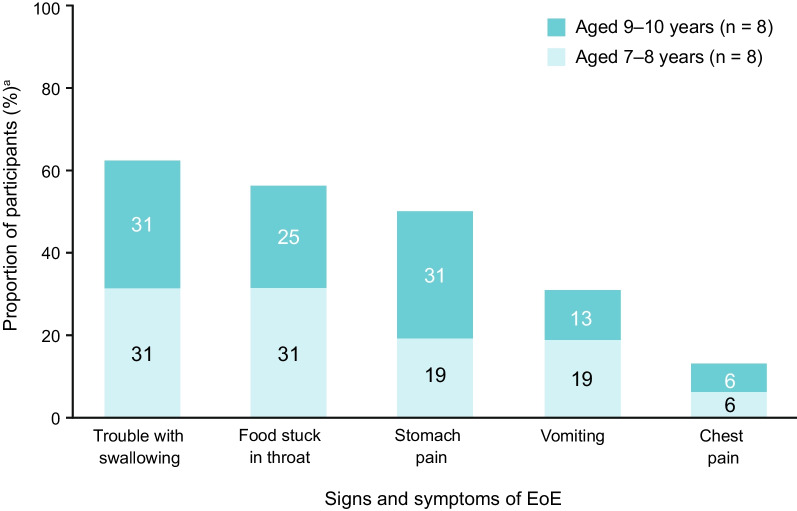
Signs and symptoms of EoE reported by participants during concept elicitation. ^a^Proportion of all participants enrolled in the study (N = 16). *EoE* Eeosinophilic esophagitis

During concept elicitation, several concepts relating to the signs and symptoms of EoE were identified (Fig. 2). The most common patient-reported concept was trouble with swallowing (overall: 63% [10/16]; 7–8 years: 63% [5/8]; 9–10 years: 63% [5/8]), followed by food getting stuck in the throat (overall: 56% [9/16]; 7–8 years: 63% [5/8]; 9–10 years: 50% [4/8]), stomach pain (overall: 50% [8/16]; 7–8 years: 38% [3/8]; 9–10 years: 63% [5/8]), vomiting (overall: 31% [5/16]; 7–8 years: 38% [3/8]; 9–10 years: 25% [2/8]), and chest pain (overall: 13% [2/16]; 7–8 years: 13% [1/8]; 9–10 years: 13% [1/8]). Concept saturation, the point at which no new signs or symptoms of EoE were reported by the participants [20, 21], was achieved after the fifth interview.

### Content validity and comprehensibility of the DSQ

#### Content validity

Most participants who provided input stated that the DSQ questions were ‘easy to complete’ (overall: Q1, 88% [14/16]; Q2, 88% [14/16]; Q3, 69% [11/16]; Q4, 69% [9/13]) and ‘relevant to someone with EoE and dysphagia’ (overall: Q1, 83% [10/12]; Q2, 92% [11/12]; Q3, 100% [9/9]; Q4, data not collected). Most participants reported that they understood the meaning of all four questions and associated responses, with greater understanding typically reported by participants aged 9–10 years than by those aged 7–8 years (Table 2). However, participants’ responses did not consistently show comprehension; some participants answered that they understood the question, but further probing suggested that they had misunderstood some of the concepts. Table 2 demonstrates the difficulties with comprehension and word fluency for each of the questions within the DSQ. For example, in Q1, some participants did not consider “pancakes” or “cereal” to be solid food because it was “fluffy and not hard” or “it got all soggy”. Participants reported that challenging concepts included ‘solid food’ (Q1, 13% [2/16]), ‘trouble swallowing’ (Q2, 13% [2/16]), ‘vomit’ (Q3, 23% [3/13]), and ‘relief’ (Q3, 6% [1/16]). One participant also reported that words such as ‘concerns’ or ‘experienced’ were difficult to understand (Q4, 33% [1/3]), and another reported that the wording of some questions was too long (Q3, 6% [1/16]; Q4, 33% [1/3]) (Table 2). Four participants reported difficulty understanding Q4 (31% [4/13]); some participants struggled to differentiate between ‘mild’, ‘moderate’, ‘severe’, and ‘very severe’ pain. Difficulties with comprehension were generally more common in participants aged 7–8 years than in those aged 9–10 years.

**Table 2:**
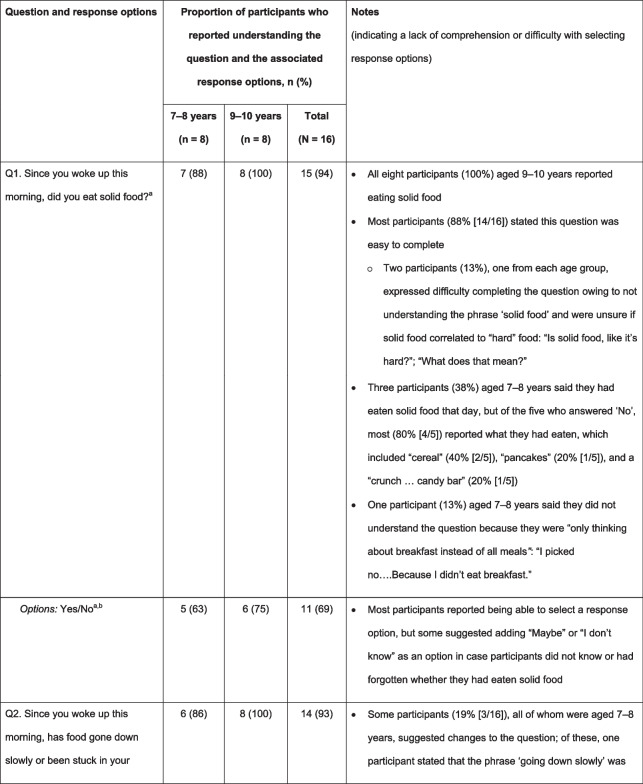

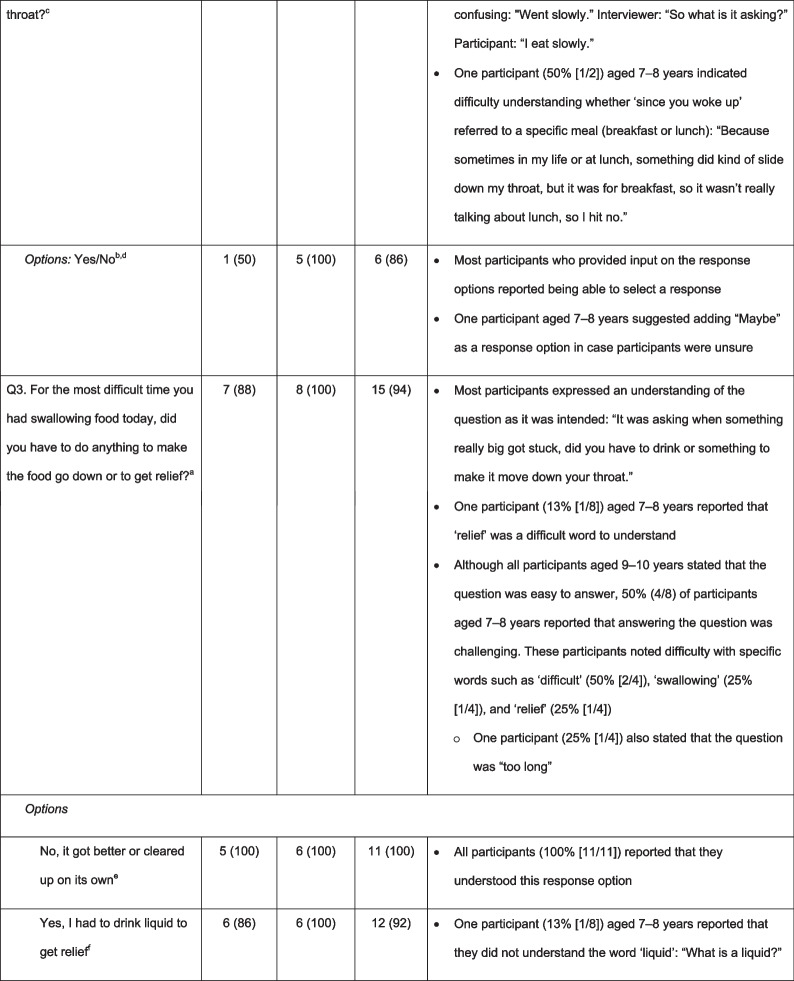

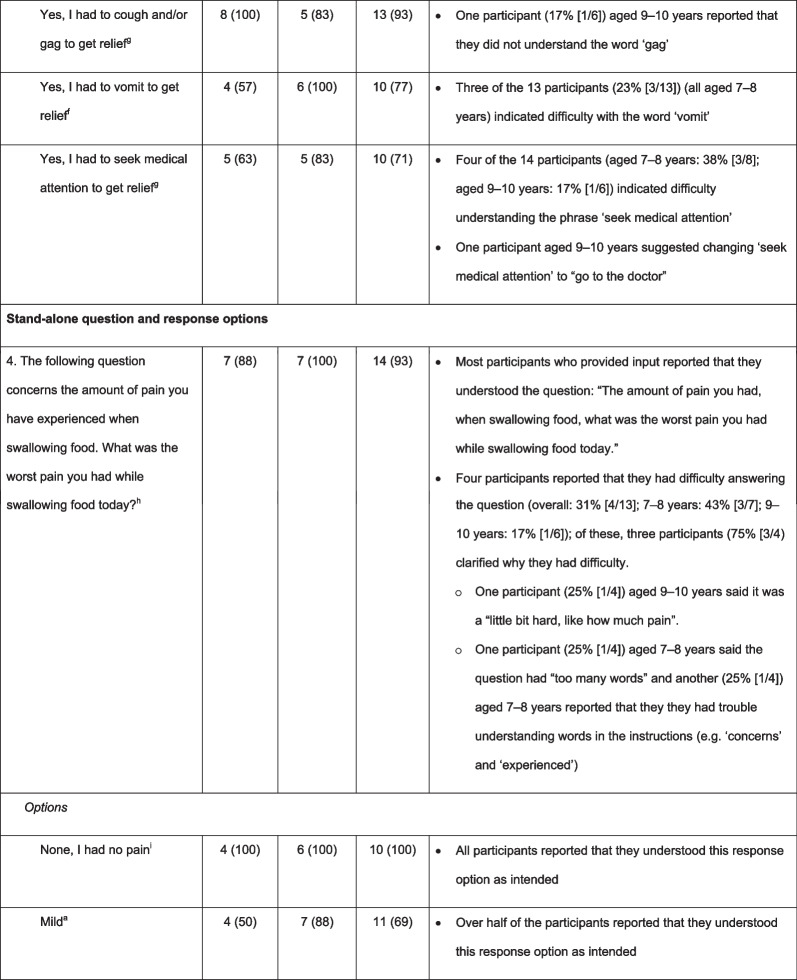

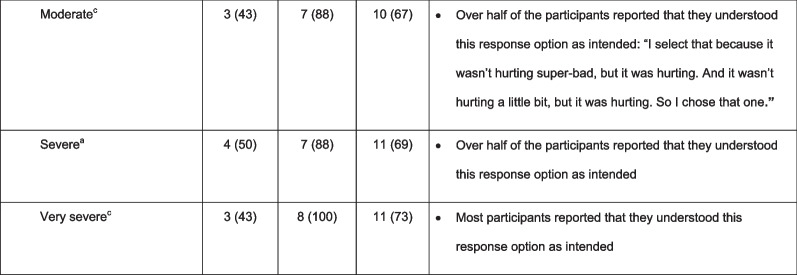
Participants who reported understanding the DSQ questions and associated response options, and notes regarding comprehension

^a^All participants responded

^b^Yes/No refers to the response options available to participants; n (%) refers to the proportion of participants who reported understanding the DSQ questions and associated response options

^c^15/16 participants responded (aged 7–8 years: n = 7; aged 9–10 years: n = 8)

^d^7/16 participants responded (aged 7–8 years: n = 2; aged 9–10 years: n = 5)

^e^11/16 participants responded (aged 7–8 years: n = 5; aged 9–10 years: n = 6)

^f^13/16 participants responded (aged 7–8 years: n = 7; aged 9–10 years: n = 6)

^g^14/16 participants responded (aged 7–8 years: n = 8; aged 9–10 years: n = 6)

^h^15/16 participants responded (aged 7–8 years: n = 8; aged 9–10 years: n = 7)

^i^10/16 participants responded (aged 7–8 years: n = 4; aged 9–10 years: n = 6)

*DSQ* Dysphagia symptom questionnaire, *Q* question

#### DSQ scores

Although most caregivers (94% [15/16]) reported that their child had experienced difficulty with swallowing, out of a possible DSQ score of between 0 and 84, the participant-reported mean (SD) score over the study period was low (overall: 7.3 [7.4]; 7–8 years: 9.2 [5.0]; 9–10 years: 6.6 [8.2]) (Table 3), suggesting infrequent and mild dysphagia. Eleven participants (69% [11/16]) completed the DSQ diary on at least 8 days during the 2-week study period, allowing DSQ scores to be calculated (Additional file 1: Table S1). Five participants were excluded from DSQ score calculations owing to having fewer than 8 days of daily diary completion, or for answering ‘No’ to Q1 on more than 5 days over the 2-week study period. For the frequency distribution of responses to the questions of the DSQ over the 2-week study period, almost half of the responses to Q2 were ‘No' (overall: 43% [90/210]; 7–8 years: 32% [33/102]; 9–10 years: 53% [57/108]), indicating that the participant did not experience dysphagia.

**Table 3:** Self-reported DSQ scores for participants aged 7–10 years with EoE

DSQ scores^a^	7–8 years (n = 8)^b^	9–10 years (n = 8)	Total (N = 16)
Mean (SD)	9.2 (5.0)	6.6 (8.2)	7.3 (7.4)
Median (range)	11.2 (3.5–12.9)	4.2 (0.0–22.6)	8.4 (0.0–22.6)

During the 2-week study period, 43% of responses to Q2, which asked whether participants had experienced dysphagia on most days, were ‘No’

^a^DSQ scores are calculated from combined responses to Q2 and Q3

^b^Five participants were excluded in the 7–8-year group for having fewer than eight completed daily diary entries (n = 4) or answering ‘No’ to Q1 on more than 5 days over the 2-week study period (n = 1)

*DSQ* Dysphagia Symptom Questionnaire, *Q* Question, *SD* Standard deviation

#### Usability of the DSQ on an electronic handheld device

Of the 15 participants who attended visit 2, most reported that completing the DSQ using the electronic handheld device was easy (overall: 93% [14/15]; aged 7–8 years: 86% [6/7]; aged 9–10 years: 100% [8/8]). One participant in the younger age group reported having a technical issue with the electronic handheld device, which prevented them from completing a few days of the diary. Overall, participants liked the electronic handheld device for completing the DSQ (100% [10/10]) and were willing to complete the DSQ every day (100% [15/15]). Most participants (85% [11/13]) reported that they would be willing to complete the DSQ for longer than a 2-week period. The median (range) number of consecutive days that the DSQ was completed was 7 (2–13) days. All participants missed at least 1 day of the DSQ (100% [16/16]) and most (67% [10/15]) reported missing a few days. One participant missed every diary entry; out of a possible 210 diary entries across the remaining 15 participants, 55 entries (26%) were missed over the 2-week study period, and each participant missed a median (range) of 3 (1–8) days of the DSQ. The reasons for not completing the DSQ were travel (20% [3/15]), forgetting to complete it (20% [3/15]), falling asleep early (13% [2/15]), and the device not being charged (7% [1/15]) or not turning on (7% [1/15]). Five participants did not provide a reason for not completing the DSQ. Feedback from caregivers indicated that participants did not experience difficulty with the device (90% [9/10]) and that they could easily manage the recompletion of the diary each day (100% [11/11]).

## Discussion

The DSQ has been shown to be a valid and reliable measure of dysphagia in patients aged 11–40 years with EoE [6, 7]; however, it has not been evaluated in children aged younger than 11 years. The aim of the current study was to investigate the content validity including comprehensibility of the DSQ in children aged 7–10 years with EoE, and the usability of the DSQ on an electronic handheld device.

Dysphagia is considered a less common symptom in children with EoE than in adolescents and adults with EoE [4, 5]. DSQ scores in our study indicated that the frequency of dysphagia was varied between the caregiver and patient-reported perspective; 43% of all participants reported no dysphagia over the 2-week study period and there was variation in the severity of dysphagia reported. Findings from a previous study suggested that there is a good correlation between patient and caregiver reports of symptoms for pediatric patients with EoE [22], which raises the question of why, during our study, the participant-reported DSQ scores in the younger patient population (aged 7–8 years) did not match caregiver reports of dysphagia frequency. This discrepancy could be because dysphagia is not an observable symptom, unlike vomiting; therefore, caregivers might consider this symptom more frequent than actually experienced by the patients. In a previous study that investigated the caregiver and patient-reported perspective for symptoms in children undergoing cancer treatment, caregivers consistently overestimated symptoms compared with self-reports from children [23]. Further investigations into the average frequency of dysphagia in this population are therefore important.

Most participants stated that the questions asked in the DSQ were relevant to someone with EoE and dysphagia. During the concept elicitation stage of the study, the most common patient-reported signs and symptoms were also covered in the DSQ. However, some participants mentioned experiencing stomach and chest pain, which are not items included as response options in question 3 of the DSQ. These responses suggest that the DSQ may not be sufficiently comprehensive for this population, and should be adapted for patients aged 7–10 years with EoE.

Although participants reported understanding the DSQ, further probing highlighted that the language may have been unsuitable for some children who did not understand some of the terms used, particularly in the younger age group (aged 7–8 years). These children often reported favorably with regard to their understanding of the DSQ, but revealed a lack of comprehension when questioned further about specific concepts and terms. For example, some participants answered ‘No’ when asked if they had eaten solid foods, but later reported that they had eaten “pancakes” or “cereal”, demonstrating a misunderstanding of the question. Overall, although more children in the older age group (aged 9–10 years) reported understanding the questions and responses than children in the younger age group, all children encountered some challenges with comprehension when completing the DSQ. These results could suggest that dysphagia in this patient population might not have been accurately reported by participants. However, most caregivers reported that their child experienced dysphagia, suggesting that dysphagia could be considered a common symptom of EoE, even in children aged as young as 7 years. These findings could therefore have implications for diagnostic protocols and therapeutic programs.

It is not uncommon for existing PRO measures to be developed in one population and assessed for use in another applicable population; for example, the EXACT-Respiratory Symptoms scale was developed for use in patients with chronic obstructive pulmonary disease and was subsequently evaluated for use in patients with idiopathic pulmonary fibrosis [24–26]. Other PRO measures have multiple versions that are age-specific; for example, the Haemo-QoL is used to evaluate the quality of life of patients with hemophilia and has separate versions that have been adapted for use in patients aged 4–7, 8–12, and 13–16 years [27]. However, to our knowledge, there are no studies in pediatric patients with EoE that have assessed the content validity of a PRO that was originally designed for adolescents and adults. The DSQ was initially developed for adolescents and adults with EoE [7]; this PRO was psychometrically validated in patients aged 11–40 years with EoE [6] before being evaluated in children aged 7–10 years with EoE during our study. Although the qualitative data collected during our study support the concepts covered by the DSQ, the phrasing of questions was somewhat problematic in this population. One participant suggested that some questions were too long. More participants in the younger age group than in the older age group expressed that they did not know the meaning of terms such as ‘concerns’, ‘experienced’, ‘vomit’, or ‘relief’. The findings of our study demonstrate that transitioning the DSQ from use in adults to use in children poses specific challenges, particularly in regard to reading level, comprehension, and vernacular in our target population. In future, rephrasing the wording of the questions in the DSQ would be important to help with comprehensibility in our population.

In other areas of research, PRO entries for 3 consecutive days is considered sufficient for analysis [28]. This is in contrast to the scoring approach that is universally used for the validated DSQ, for which a 14-day recall period is used and a minimum of eight diary entries are required to calculate the DSQ score [6]. Most participants in our study would have met the threshold of 3 consecutive days of PRO entries; however, 19% (3/16) would still not have had enough consecutive days of data to calculate a DSQ score. Additionally, most participants missed at least 1 day of the DSQ diary. Furthermore, we anticipate that for PROs in EoE, 2 complete weeks of data are likely needed for objective symptom assessment. It has been suggested that the typical duration for symptom diary completion when continuous data recording is required is 2–4 weeks [29]. Additionally, there is evidence that errors in recall are more likely to occur when the time frame exceeds 1 week [29], supporting the daily completion of symptom diaries.

One limitation of this study is that the questions and response options in the DSQ were not modified to improve comprehensibility in children aged 7–10 years. For future versions of the DSQ, changing the wording for certain questions and response options so it is more accessible for a younger population should be considered. For example, in the cases of ‘vomit’, ‘relief’, or ‘seek medical attention’, more appropriate phrases such as “puke”, “feel better”, or “go to a doctor”, respectively, were suggested by participants or their caregivers. Alternative wording choices could also be explored. Another limitation is that the sample size was small, considering the increasing incidence and prevalence of EoE as well as its substantial heterogeneity [30]. However, with the qualitative nature of the study design and the interviews conducted, concept saturation was achieved. The results also may not be representative of other geographic regions in the USA, because this study was conducted in only two US cities; future studies could therefore seek to assess the DSQ in a larger population across multiple geographic locations. The final analysis was not shared with participants for feedback (member checking), owing to their age (7–10 years). However, attempts were made to validate the participants’ responses during the interviews using probing questions. A final limitation is that for participants recruited through CURED, the diagnosis of EoE was based on a caregiver report of a clinically confirmed diagnosis, rather than confirmation via medical records.

A strength of this study is that it was a qualitative, noninterventional study and the population of interest recruited was generally representative of the population of patients with EoE in terms of sex and race [31]. Additionally, unlike other studies that have investigated novel symptom questionnaires for children with EoE [32], our study evaluated the content validity, including comprehensibility, of an existing PRO measure and its usability in a pediatric population of patients with EoE. Different data collection modes (research triangulation) were used; data were analyzed qualitatively and quantitatively to increase the credibility of the research findings.

## Conclusions

These findings suggest that the DSQ may not be sufficiently comprehensive for use in children aged 7–10 years with EoE. However, following modifications to the wording and phrasing to improve comprehensibility and comprehensiveness, particularly for children aged 7–8 years, the DSQ could be modified to create a bespoke tool that is appropriate for this patient population. Educating children and caregivers on how to use the DSQ would be necessary before its use, to ensure full understanding of the questions and response options and to emphasize the importance of daily diary entry. Finally, given that most participants experienced difficulty swallowing, which was a somewhat unexpected finding in this younger cohort of patients with EoE, further studies assessing dysphagia in this age group are needed, because this could have important implications for clinical trials in this population.

### Supplementary Information

**Additional file 1:** Supplementary material.

## Data Availability

The data sets, qualitative interview transcripts, and case report forms are not publicly available because the content is being used for strategic research development. The data may be shared upon request; please contact the corresponding author.

## Abbreviations

CURED: Campaign Urging Research for Eosinophilic Disease
DSQ: Dysphagia Symptom Questionnaire
EoE: Eosinophilic esophagitis
FDA: Food and Drug Administration
PRO: Patient-reported outcome
Q1: Question 1
Q2: Question 2
Q3: Question 3
Q4: Question 4
SD: Standard deviation
