# Supplementary material for: Qualitative assessment of the suitability of the Dysphagia Symptom Questionnaire to monitor dysphagia in children aged 7–10 years with eosinophilic esophagitis
Source: J Patient Rep Outcomes. 2023 Oct 31;7:110. doi: 10.1186/s41687-023-00646-z (PMC10618132; doi:10.1186/s41687-023-00646-z)
Supplement: Supplementary file 1 — Additional file 1: Supplementary material. [file 41687_2023_646_MOESM1_ESM.docx]

**Supplementary material**

**Supplementary methods**

The cognitive interview guide is provided below, which was used as a guide only. The actual areas of conversation might have been fluid and could have been discussed with flexibility in the sequence of probes and discussion. The interview lasted approximately 60 min with breaks as needed. The interviewer was allowed to adapt the guide so topics were covered in the amount of time allotted for the session or to best elicit responses from the participants.

Probes included in this script could have been used to guide the discussion if the participant seemed confused or was unable to provide a response without assistance. Additional unscripted probes to be used to gain further information or clarification may include:

• clarification: I don’t quite understand that;

• expressing understanding: How do/how would you cope with that?;

• justification: What makes you say that?;

• importance: I’m not sure how these two things are linked;

• extending narrative: Tell me a bit more about that;

• accuracy: Let’s see if I’ve got that right.

INTRODUCTION (Both Caregiver and Child)

Thank you for being a part of our study. My name is [X]. Let me tell you a little about what we are going to do today. [Child’s name], if you agree to be in the study, I will ask a few questions about your EoE, which may include questions about food getting stuck in your throat or food going down slowly. After that, I will ask you to fill out a questionnaire. There are no right or wrong answers to the questionnaire, we want to learn your opinions. Then, I’ll ask you some questions about the questionnaire to find out what you think of it.

Before we get started, I want to review the forms labeled Informed Consent Form and Assent Form. [Caregiver], the consent form is for you to review and ask any questions you may have on the study. If you consent to your child’s participation, please complete the forms by providing your initials and/or full signature where indicated on the bottom of each page. [Name of child], you will be reading the Assent Form. If you want to take part in this study, please read this form and let me know if you have any questions.

Allow the caregiver and child as much time as needed to review the consent/assent forms, and answer any questions. Will it be ok for me to audio record this interview [directed to the caregiver]? If they agree to participate, tell /show them where to sign and initial the forms.

Caregiver will then be asked to complete the Sociodemographic Questionnaire.

Remind caregiver he/she is welcome to stay for the interview, but we would like to hear from the child about their impressions of the questionnaire. I am really interested in what [Name of child] has to say. Is this okay with both of you?

Begin Recorder: This is study EVA-21524-01, this is [Participant ID] and today’s date is [insert date]. Before we start, do you have any questions [directed to both the child and caregiver]? I want to confirm that you read and signed the consent form [directed to the caregiver]. We will give you a copy to take home.

Will it be ok for me to audio record this interview [directed to the caregiver]? Your child’s name will not be linked with your responses in any way. The recording will be used to help us write a report about the things we talked about here today. The report also will include information that we get from other children and or caregivers that were in this study.

Thank you for being in our study. The reason we are doing this study is to talk to children who have EoE to find out what you think about a new questionnaire. I want to remind you that you are a volunteer and this means that you can skip any question you don’t want to answer; and you can stop the interview at any time. Your responses will be kept as confidential as possible. The only people that may look at your responses are those we reviewed in the assent form.

Also, I want to tell you that I am not a doctor or nurse, so if you have any medical questions that come up because of our discussion today, please ask your [mom/dad/doctor].

Part I. Concept Elicitation

I want to start by asking you a few general questions about yourself.

1. Ice breaker question [favorite character, color, place to visit]
2. How old are you?
3. What kind of food do you like to eat? [icebreaker]
4. What food is harder to swallow than other food?
5. What food is easier to swallow than other food?
6. What kinds of foods are “solid foods”? Can you give me some examples?
7. Do you eat that food?

a. If no/if limited: Why not? Can you tell me more about that?

b. Do you have any trouble swallowing that kind of food?

1. Are there any “solid foods” that you don’t eat?

a. Probe for each food example:

b. What foods?

c. Why don’t you eat that food? Probe for relation to EoE or just don’t want/don’t like the food.

d. How often do you have to avoid/not eat that food?

1. Do you ever feel that you have trouble or difficulty swallowing?
2. Do you ever feel that food gets stuck or goes down slowly in your throat?
3. What does trouble swallowing mean to you?
4. Is there a name for the condition you have that can make it [interviewer will use the child’s terminology for difficulty swallowing]?
5. Do you know how old you were when a doctor first told you and your family you had [use participant terminology]? How old were you?
6. What bothers you the most about [use participant terminology] Probe symptom then impact experience.
7. Any others? Can you tell me what happens when you experience that?

Part II. Cognitive Interview of DSQ Diary

Thanks. Now we will go on to the questionnaire. First, I’m going to show you how this device works and how to get to the questionnaire. [Train participant on use of the electronic device]

I’m going to ask you now to fill out a questionnaire called the Dysphagia Symptom Questionnaire or DSQ. Please read the instructions and then answer the questions. Please remember there are no right or wrong answers. If you get stuck on a word, just ask for help, ok? Feel free to take as much time as you need. Please let me know when you are done and I will ask you some follow-up questions.

Record comments from participant while completing questionnaire, assist participant as needed to ensure questionnaire is fully completed. When participant is done completing the items: Now I would like to ask you some things about the questionnaire you just completed.

Now I’m going to interview you about the questionnaire that you just finished. This will help us understand if kids will be able to fill it out by themselves. Ok? We just want to know what you think so please feel free to give me your advice, you’re the expert!

Instructions

1. What are the instructions asking you to do? What do they mean to you?

a. Probe if needed: In general, what were you thinking about when you read these instructions?

2. Was there anything hard to understand about the instructions? Were any words difficult to read or understand?

a. If yes: Please tell me about any suggestions you may have to make these instructions better or easier to understand (e.g., please tell me if there are any words or phrases you would change).

***Items:*** *Now let’s take a closer look at each of the questions you read and answered. I want you to know that some of my questions are going to seem repetitive. However, I need to do this because it’s important for me to know how you understand each question so the research team can decide if we need to make changes or make something easier to understand.*

1. Question 1: Since you woke up this morning, did you eat solid food?
2. In your own words, what is this question asking you?
3. What answer did you choose [*probe*: “yes,” “no”] and why?
4. *Probe:* What experiences were you thinking about when answering this question?
5. Was this question hard for you to understand? If yes, how would you make it easier to understand?
6. *Probe (if any changes are suggested):* What would you change, and why?
7. Is this a good or bad question to ask someone with EoE? Why do you say that?

**Response Options:** *I would now like for you to look at the response options for question one.*

1. Could you choose an answer for the question using the answer choices listed?
2. If “No,” How come?
3. *Probe:* Were there not enough choices?
4. If you avoided solid food during the day because of something other than EoE, how would you have answered that question? *If not already probed in interview, ask about avoiding food due to EoE and due to other reasons (rationale, frequency, etc.)*

*Let’s move on to question two.*

1. Question 2: Since you woke up this morning, has food gone down slowly or been stuck in your throat?
2. In your own words, what is this question asking you?
3. What answer did you choose [probe: “yes,” “no”] and why?
4. *Probe:* What experiences were you thinking about when answering this question?
5. Was this question hard for you to understand? If yes, how would you make it easier to understand?
6. *Probe (if any changes are suggested):* What would you change, and why?
7. Is this a good or bad question to ask children with difficulty swallowing? Why do you say that?
8. Could you choose an answer for the question using the answer choices listed?
9. If “No,” How come?
10. *Probe:* Were there not enough choices?

*Let’s move on to question three.*

1. Question 3: For the most difficult time you had swallowing food today, did you have to do anything to make the food go down or to get relief?
2. In your own words, what is this question asking you?
3. If needed, work with the participant to break-down the question. Thinking about the most difficult time you had to swallow food today; how was it difficult?
4. Did you have to do anything to help get the food down?
5. What answer did you choose [probe: “yes,” “no”] and why?
6. *Probe:* What experiences were you thinking about when answering this question?
7. Was this question hard for you to understand? If yes, how would you make it easier to understand?
8. *Probe (if any changes are suggested):* What would you change, and why?
9. Is this a good or bad question to ask children with difficulty swallowing? Why do you say that?

**Response Options:** *I would now like for you to look at the response options for question three.*

1. Could you choose an answer for the question using the answer choices listed?
2. If “No,” How come?
3. *Probe:* Were there not enough choices?
4. Is it hard to tell the difference between any of these response choices? Why or why not?
5. Using your own words, what does “No, it got better or cleared up on its own” mean to you?
6. Using your own words, what does “I had to drink liquid to get relief” mean to you?
7. Using your own words, what does “I had to cough and/or gag to get relief” mean to you?
8. Using your own words, what does “I had to vomit to get relief” mean to you?
9. Using your own words, what does “I had to seek medical attention to get relief” mean to you?
10. Here are cards with the answers on each. Can you put them in order from the best to the worst? (Present flash cards with written response option on each; use terms that are age-appropriate like ‘best’ and ‘worst’ but indicate it’s the severity.)

*Let’s move on to question four.*

1. *Question 4:* The following question concerns the amount of pain you have experienced when swallowing food. What was the worst pain you had while swallowing food today?
2. In your own words, what is this question asking you?
3. What answer did you choose [probe: “yes,” “no”] and why?
4. *Probe:* What experiences were you thinking about when answering this question?
5. Was this question hard for you to understand? If yes, how would you make it easier to understand?
6. *Probe (if any changes are suggested):* What would you change, and why?
7. Is this a good or bad question to ask children with difficulty swallowing? Why do you say that?

**Response Options:** *I would now like for you to look at the response options for question four.*

1. Could you choose an answer for the question using the answer choices listed?
2. If “No,” How come?
3. *Probe:* Were there not enough choices?
4. Is it hard to tell the difference between any of these response choices? Why or why not?
5. Using your own words, what does “None, I had no pain” mean to you?
6. Using your own words, what does “Mild” mean to you?
7. Using your own words, what does “Moderate” mean to you?
8. Using your own words, what does “Severe” mean to you?
9. Using your own words, what does “Very Severe” mean to you?

**Final Impressions**

1. What do you think of the questionnaire?
2. Do you think the questions make sense to ask kids who have difficulty swallowing? (e.g., do the questions apply to you?)
3. If not, which ones do not make sense to you?
4. Why do you think they don’t they don’t make sense for kids with difficulty swallowing?
5. After talking about things more, are there any questions that we asked you that aren’t good ones to ask about for difficulty swallowing?
6. If, yes, which one(s)?
7. Why do you think they aren’t good to ask?
8. Are there any questions that don’t apply to you?
9. If, yes, which one(s)?
10. Are there any questions that we should have asked that we did not?
11. Yes? Which types of activities or things should we ask about?

**End of Interview**

Thank you for your help. We have completed the interview.

**DSQ Diary Usability and Feasibility Follow-up Call**(completed approximately 2 weeks after the initial interview)

**INTRODUCTION (Both Caregiver and Child)**

*Hi [child’s name]. My name is [X] and we met a couple weeks ago. Thank you for being a part of our research study. Let me tell you a little about what we are going to do today. This will be a very short call. I just want to ask you a few questions about your experience completing the questionnaire for the last two weeks. If it’s OK with you, I’m going to audio record this interview too. Do you have any questions before we begin? Answer any questions.*

***Begin Recorder:*** *This is study EVA-21524-01, this is [Participant ID] and today’s date is [insert date]. I want to confirm that it is ok for me to audio record this interview? Wait for verbal response from caregiver.*

**Part III. DSQ Diary Usability and Feasibility**

1. What did you think about answering the questionnaire every day*?*
2. Were there any days you had to skip? If so, why?
3. Did you have any difficulty remembering to complete the questionnaire every day?
4. What could we do to make remembering to complete the questionnaire easier?
5. What did you think about using the electronic device to answer the questionnaire every day?
6. Did you have any issues with the electronic device?
7. If so, what were they?
8. Would you be willing to complete a questionnaire like this every day?
9. What’s the longest amount of time you’d be willing to complete the questionnaire?

**End of Interview**

Thank you for your help. We have completed the interview.

**Supplementary tables**

**Table S1.** The Dysphagia Symptom Questionnaire (DSQ, version 4.0) and scores for each response option^a,b^

| **Question** | **Response options** | **Score** |
| --- | --- | --- |
| 1. Since you woke up this morning, did you eat solid food?^c^ | No | – |
|  | Yes | – |
| 1. Since you woke up this morning, has food gone down slowly or been stuck in your throat? | No | 0 |
|  | Yes | 2 |
| 1. For the most difficult time you had swallowing food today, did you have to do anything to make the food go down or to get relief? | No, it got better or cleared up on its own | 0 |
|  | Yes, I had to drink liquid to get relief | 1 |
|  | Yes, I had to cough and/or gag to get relief | 2 |
|  | Yes, I had to vomit to get relief | 3 |
|  | Yes, I had to seek medical attention to get relief | 4 |
| 1. The following question concerns the amount of pain you have experienced when swallowing food. What was the worst pain you had while swallowing food today?^d^ | None, I had no pain | 0 |
|  | Mild | 1 |
|  | Moderate | 2 |
|  | Severe | 3 |
|  | Very severe | 4 |

Adapted from Hudgens S et al (2017) J Patient Reported Outcomes 1:3, distributed under the terms of the Creative Commons Attribution 4.0 International License (<http://creativecommons.org/licenses/by/4.0/>) with minor editorial changes [1]

^a^The scoring algorithm was constructed from responses to Q2 and Q3 to ensure that the final DSQ score represented the frequency and severity of dysphagia. DSQ scores were calculated using the following equation [1]:

$$\text{DSQ score = }\frac{\left( \text{sum of points from questions}\text{ 2}\text{ and }\text{3}\text{ of the daily DSQ diary} \right)\text{ }\text{×}\text{ }\text{14}\text{ days}}{\text{number of diary days reported with non-missing data}}$$

^b^Participants needed to have completed the diary for at least 8 days during the 2-week study period for DSQ scores to be calculated [1]

^c^Responses to Q1 were not used to calculate DSQ scores

^d^Q4 is a standalone item on the DSQ [1]

*DSQ* Dysphagia Symptom Questionnaire, *Q* Question

**Table S2.** Baseline demographics of caregivers of children with EoE

| **Demographics** | **Caregivers of children with EoE** | | |
| --- | --- | --- | --- |
|  | **7–8 years**  **(n = 8)** | **9–10 years**  **(n = 8)** | **Total (N = 16)** |
| Age, years | | | |
| Mean (SD) | 34.6 (7.2) | 39.6 (6.1) | 37.1 (6.9) |
| Median (range) | 35.0 (24.0–44.0) | 37.5 (33.0–49.0) | 36.5 (24.0–49.0) |
| Sex | | | |
| Male | 0 (0) | 1 (13) | 1 (6) |
| Female | 8 (100) | 7 (88) | 15 (94) |
| Ethnicity | | | |
| Hispanic or Latino | 0 (0) | 1 (13) | 1 (6) |
| Not Hispanic or Latino | 8 (100) | 7 (88) | 15 (94) |
| Race | | | |
| White | 7 (88) | 4 (50) | 11 (69) |
| Black or African American | 1 (13) | 2 (25) | 3 (19) |
| Other^a^ | 0 (0) | 2 (25) | 2 (13) |
| Marital status | | | |
| Married | 6 (75) | 7 (88) | 13 (81) |
| Single | 1 (13) | 0 (0) | 1 (6) |
| Divorced/separated | 0 (0) | 1 (13) | 1 (6) |
| In a relationship, not married | 1 (13) | 0 (0) | 1 (6) |
| Employment status | | | |
| Full-time | 2 (25) | 2 (25) | 4 (25) |
| Part-time | 2 (25) | 2 (25) | 4 (25) |
| Homemaker | 1 (13) | 1 (13) | 2 (13) |
| Student | 0 (0) | 1 (13) | 1 (6) |
| Not working | 3 (38) | 2 (25) | 5 (31) |
| Education level | | | |
| Associate degree, vocational, technical or trade school | 1 (13) | 1 (13) | 2 (13) |
| Some college (< 1 year) | 1 (13) | 0 (0) | 1 (6) |
| Some college (2–3 years) | 2 (25) | 0 (0) | 2 (13) |
| University/college degree | 3 (38) | 4 (50) | 7 (44) |
| Postgraduate degree | 1 (13) | 3 (38) | 4 (25) |
| Relationship to the child with EoE | | | |
| Mother/stepmother | 8 (100) | 7 (88) | 15 (94) |
| Father/stepfather | 0 (0) | 1 (13) | 1 (6) |
| Immediate family members with EoE | | | |
| Yes | 1 (13) | 4 (50) | 5 (31) |
| No | 6 (75) | 4 (50) | 10 (63) |
| Unsure | 1 (13) | 0 (0) | 1 (6) |

All data are reported as n (%), unless otherwise stated

^a^Other race includes: ‘Latina’ (n = 1) and ‘white and Hispanic’ (n = 1)

*EoE* Eosinophilic esophagitis, *SD* Standard deviation

**Reference**

1. Hudgens S, Evans C, Philips E, Hill M (2017) Psychometric validation of the Dysphagia Symptom Questionnaire in patients with eosinophilic esophagitis treated with oral budesonide suspension. J Patient Rep Outcomes 1:3.
